# Extracellular Matrix and Cellular Plasticity in Musculoskeletal Development

**DOI:** 10.3389/fcell.2020.00781

**Published:** 2020-08-25

**Authors:** Sophia Ka Yan Ma, Andy Shing Fung Chan, Aqsa Rubab, Wilson Cheuk Wing Chan, Danny Chan

**Affiliations:** ^1^School of Biomedical Sciences, The University of Hong Kong, Hong Kong, China; ^2^Department of Orthopedics Surgery and Traumatology, The University of Hong Kong-Shenzhen Hospital, Shenzhen, China; ^3^The University of Hong Kong Shenzhen Institute of Research and Innovation (HKU-SIRI), Shenzhen, China

**Keywords:** extracellular matrix, plasticity, development, limb regeneration, chondrocyte, hypertrophic chondrocyte, joint formation, muscle

## Abstract

Cellular plasticity refers to the ability of cell fates to be reprogrammed given the proper signals, allowing for dedifferentiation or transdifferentiation into different cell fates. *In vitro*, this can be induced through direct activation of gene expression, however this process does not naturally occur *in vivo*. Instead, the microenvironment consisting of the extracellular matrix (ECM) and signaling factors, directs the signals presented to cells. Often the ECM is involved in regulating both biochemical and mechanical signals. In stem cell populations, this niche is necessary for maintenance and proper function of the stem cell pool. However, recent studies have demonstrated that differentiated or lineage restricted cells can exit their current state and transform into another state under different situations during development and regeneration. This may be achieved through (1) cells responding to a changing niche; (2) cells migrating and encountering a new niche; and (3) formation of a transitional niche followed by restoration of the homeostatic niche to sequentially guide cells along the regenerative process. This review focuses on examples in musculoskeletal biology, with the concept of ECM regulating cells and stem cells in development and regeneration, extending beyond the conventional concept of small population of progenitor cells, but under the right circumstances even “lineage-restricted” or differentiated cells can be reprogrammed to enter into a different fate.

## Introduction

Over the past couple of decades, cellular reprogramming or plasticity has gained traction as a means to address different areas of research, ranging from the understanding of disease progression, to manipulating cells *in vitro* (Gilbert et al., 2010; Madl et al., 2018), and as a potential target for direct regeneration (Blau and Pomerantz, 2011). The idea of cellular plasticity was first described as a concept by Helen Blau in the 1980s (Blau et al., 1985) which proposed that differentiated cells were not a terminal endpoint, and should be regarded as a cellular “state” (Zipori, 2004) that had to be actively maintained by co-ordination of both intrinsic and extrinsic factors. Cell states could therefore be altered depending on the signals they receive, changing gene expression profiles, and behavior (Blau et al., 2001; Estrov, 2009; Merrell and Stanger, 2016).

Several key studies have demonstrated this between the 1980s and early 2000s, including cloning of “Dolly the sheep” by somatic nuclear transfer (Wilmut et al., 1997), expression of muscle related genes in heterokaryons (Blau et al., 1983) and the identification of the “Yamanaka factors” to induce pluripotent stem cells from fibroblasts (Takahashi and Yamanaka, 2006). Other studies also demonstrated that trans-differentiation of mature cells into a different cell types can be achieved by one or several key transcription factors (Davis et al., 1987; Zhou et al., 2008; Ieda et al., 2010; Vierbuchen et al., 2010).

While these studies demonstrate the role of transcription factors in determining cell fate, cells independently altering their gene expression profiles does not occur naturally in living organisms. Instead, the surrounding microenvironment of cells will dictate how they respond and behave under normal physiological conditions. For stem cell populations, a highly specialized microenvironment, the stem cell niche, is composed of the extracellular matrix (ECM), signaling factors, and niche cells that provides coordinated signals to direct specific outcomes (Voog and Jones, 2010).

## The ECM Integrates Both Biochemical and Mechanical Signaling in the Stem Cell Niche

In the native environment, the role of the ECM in the stem cell niche is as important as biochemical signals. In addition to providing mechanical force, the ECM also regulates biochemical signals, as it binds and localizes signaling molecules (Wang et al., 2008; Shi et al., 2011), and presentation to cell under mechanical loading or ECM remodeling (Davis et al., 2000). Therefore, the ECM can be considered as a multifaceted component of the niche that can integrate both biochemical and mechanical cues to regulate cells.

**FIGURE 1:**
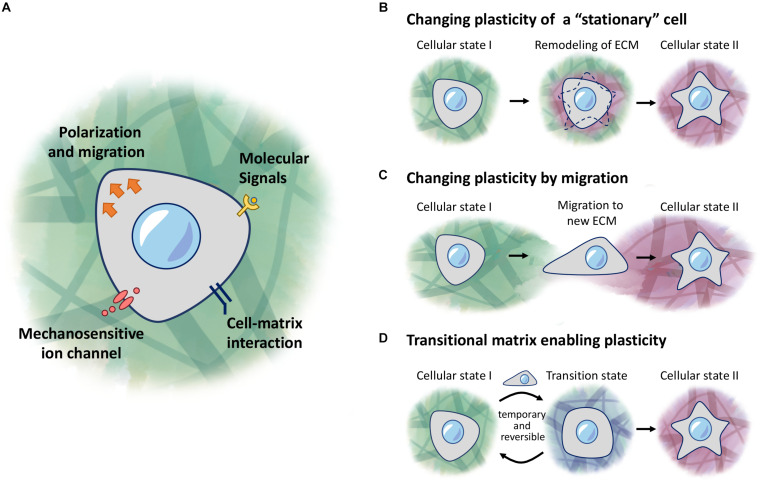
ECM regulation of cellular plasticity. **(A)** Cells respond to molecular signals and mechanical properties of the ECM through receptors and ion channels on the cell membrane. **(B–D)** Models of regulation of cellular plasticity. **(B)** Cells responding to local changes in the ECM environment to induce changes in behavior. **(C)** Cells receive new cues when migrating into a new environment. **(D)** A transitional matrix is temporarily remodeled from the homeostatic native ECM to induce changes to cellular plasticity, which then reverts back to the native ECM once the cellular process is complete.

The study by Engler et al. (2006) first highlighted the importance of mechanical force, such as matrix stiffness in directing mesenchymal stem cell differentiation, which can act independently of transcription factors. This study and others have demonstrated how the ECM, which was once regarded as a primarily structural component, can actively regulate cells through what is known as mechanotransduction (Pelham and Wang, 1997; Lo et al., 2000; McBeath et al., 2004; Gilbert et al., 2010; Wang et al., 2012; Urciuolo et al., 2013; Mao et al., 2016; Watt, 2016). Thus, mechanical forces are translated through signaling cascades, to affect changes that occur in the nucleus and gene expression. This is achieved through ECM-binding receptors such as integrins, mechanosensitive channels, G-coupled protein receptors, and growth factor receptors, which are involved in translating the various signals provided by the ECM (Figure 1A; Orr et al., 2006; Wang et al., 2009; Vining and Mooney, 2017; Jahed and Mofrad, 2019).

Furthermore, studies have shown that the structure of the actin-cytoskeleton network as a response to the outside environment can lead to enhanced reprogramming of cells. For example, reducing the stiffness of the matrix alone is sufficient to increase expression of *Oct4* and *Nanog* in HEK 293 cells without additional transcription factors (Guo et al., 2014). Moreover, combining both substrate stiffness and transcription factors can lead to an increase in euchromatic and fewer heterochromatic nuclear DNA regions, and results in enhanced iPSC conversion (Gerardo et al., 2019), indicating that alteration of chromatin state as a result of mechanical signaling can work synergistically with transcription factors that can improve the efficiency of reprogramming events. These are just a few examples of how current research are uncovering the potential of the extracellular matrix, working together with the correct combination of signaling factors as a means of directing cell fate.

## Cellular Reprogramming *in vivo*

A recent review highlighted studies of cellular reprogramming that occurs in development, or as a response to stress (Merrell and Stanger, 2016). Cells in each tissue tend to have a specialized niche specific to that tissue (Voog and Jones, 2010), and how these niches are maintained and remodeled will be context dependent. Changes to this niche will affect the outcome of cell behavior that can lead to either favorable (repair, regeneration) (Jopling et al., 2010; Mercer et al., 2013), or detrimental events such as fibrosis or degeneration (Moyer and Wagner, 2011; Bryant et al., 2017).

It is now clear that there are resident progenitor cells present in practically all mammalian tissues which serve to maintain turnover of cells during homeostasis (Voog and Jones, 2010). However, if this system is challenged in mammalians due to significant stress, it often fails to recover sufficiently, leading to permanent tissue damage (Kaur et al., 2015; Rockey et al., 2015; Talman and Ruskoaho, 2016). On the other hand, there are regenerative model organisms that are able to overcome this hurdle and effectively remodel the microenvironment to allow for complete regeneration. This can be achieved through employing cellular reprogramming, such as dedifferentiation or re-entry into the cell cycle, in order to regenerate tissues such as the heart (Jopling et al., 2010), an entire limb (Tanaka et al., 1997; Kumar et al., 2000), or any missing part of the body in planarians (Ivankovic et al., 2019). Understanding how these niches are formed and remodeled *in vivo*, and how they function under different biological contexts, will therefore provide insights into how to better develop tools needed to improve the directed-regulation of cells *in vitro* or enhance these processes *in vivo*.

**FIGURE 2:**
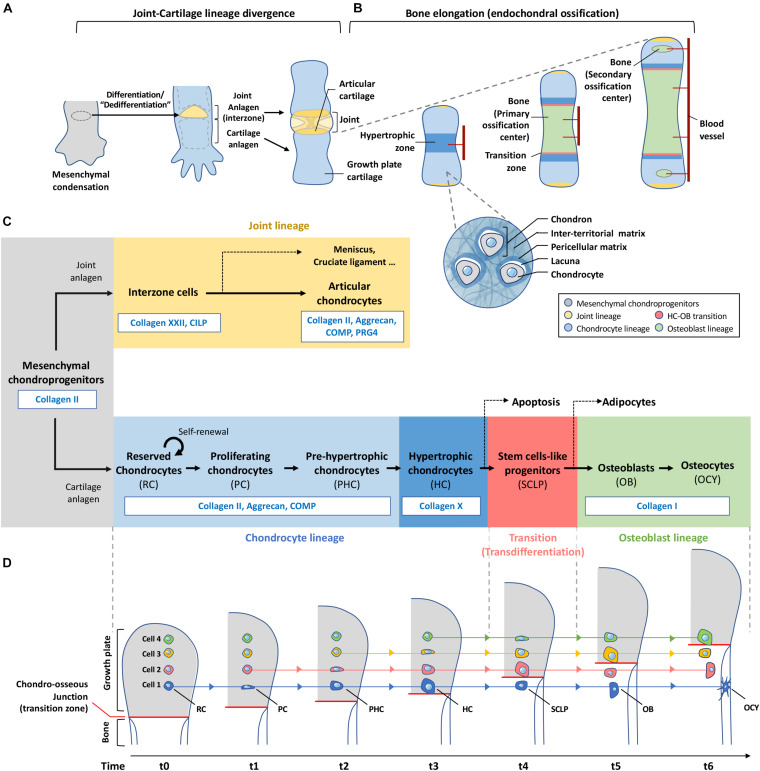
Plasticity of chondrocytes in differentiation, de-differentiation and transdifferentiation. **(A)** Mesenchymal condensation contains progenitor cells for both joint and growth plate cartilage formation. Cells at the site of future joint undergo dedifferentiation to form the interzone which eventually give rise to different joint components including the articular cartilage. **(B)** Chondroprogenitors outside the interzone become part of the growth plate cartilage and differentiate into chondrocytes which are located inside individual lacunae and bound with pericellular matrix. Each of these cocoon-like structures is called a “chondron.” Bones elongate through a developmental event called endochondral ossification. Chondrocytes at the central region of the cartilage analgen first undergo hypertrophy. Blood vessels are invaded to induce remodeling of cartilage matrix to mineralized bone matrix. Cartilage is then separated by the primary ossification center into the distal and proximal parts where the growth plates are formed. Before the end of puberty, chondrocytes in the growth plate actively undergo programmed differentiation and transdifferentiation as shown in **(C,D)**, contributing to the formation and elongation of bones. Invasion of blood vessels in-between the articular surface and the growth plates leads to hypertrophy of cells and hence formation of secondary ossification centers at bone ends of a long bone. **(C)** This roadmap shows the changes of matrix production with the differentiation status of chondroprogenitors-descendant cells. Same color scheme is applied in **(A–C)** to indicate the fate of each lineage in skeletal tissues. **(D)** Chondrocytes are not actively migrating to the ossification front during endochondral ossification. Instead, they stay at the same position receiving external cues at specific moment to differentiate and remodel its surrounding matrix. Hypertrophic chondrocytes at the chondro-osseous junction produce enzymes to remove matrix and release themselves to the bone marrow, and transit to stem-cell like progenitors for osteoblasts which will finally differentiate into osteocytes buried in bone. RC, reserved chondrocyte; PC, proliferating chondrocyte; PHC, Pre-hypertrophic chondrocyte; HC, Hypertrophic chondrocyte; SCLP, Stem cells-like progenitors; OB, Osteoblast; OCY, Osteocyte.

**FIGURE 3:**
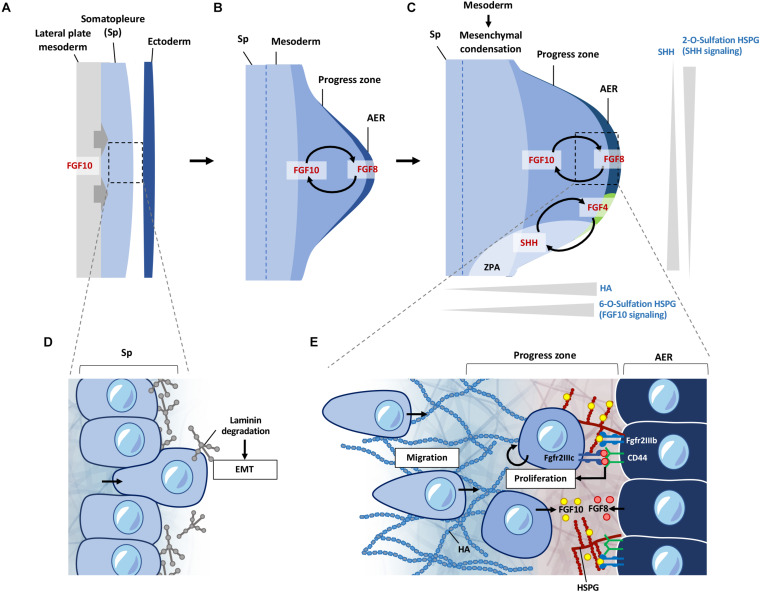
ECM regulation of signals during early limb bud formation. **(A)** FGF10 signaling from the lateral plate mesoderm induces EMT of somatopleure cells toward the ectoderm. **(B)** FGF10 induces formation of the AER from the overlying ectoderm, inducing FGF8 expression at the AER. FGF10 and FGF8 form a feed-forward signaling loop. FGF signaling induces proliferation and migration of cells in the progress zone leading to outgrowth of the limb bud. **(C)** HSPG sulfation patterns regulate diffusion of FGF and SHH signaling. 6-O sulfation regulates FGF10 binding to HSPG leading to enhanced FGF10 signaling at the progress zone/AER boundary. FGF8 first induces SHH expression at the ZPA, which is then later maintained by a feed-forward loop of FGF4-SHH signaling. 2-O sulfation of HSPG negatively regulates SHH signaling from the ZPA at the posterior regions of the limb bud, limiting diffusion of SHH at the posterior regions. HA is present at higher levels at the progress zone but absent from the AER. HA is also reduced during initiation of mesenchymal condensation in the limb bud. **(D)** Somatopleure cells are attached to laminin on the basal side. Laminin is degraded upon induction of EMT, liberating cells to migrate to form the limb bud mesenchyme. **(E)** FGF10 from the limb mesenchyme binds to 6-O sulfated HSPG and is presented to the Fgfr2b receptor on the AER cells, which induces *FGF8* to be produced by the AER cells. FGF8 secreted by the AER is bound by CD44, which presents FGF8 to the Fgfr2c receptor to cells in the progress zone, leading to proliferation of cells in the progress zone during early limb bud formation. FGF8 also induces *FGF10* expression, leading to a feed-forward loop of FGF10-FGF8 signaling. EMT, epithelial mesenchymal transition; Sp, somatopleure; AER, apical ectodermal ridge; ZPA, zone of polarizing activity; HA; hyaluronic acid; HSPG, heparan sulfate proteoglycan.

While cells can encounter different environments under various scenarios, this review will focus on three main concepts: (1) a stationary cell in a changing environment (Figure 1B), (2) a migratory cell encountering a new environment (Figure 1C), (3) and a cell in a transitional environment during tissue regeneration, and returning to homeostatic conditions upon completion (Figure 1D). In the stationary cell model, cells are responding to changes that occur in the surrounding environment, as they or other cells modify the properties of this environment, and subsequently acquire different fates as the ECM changes over time. In the second scenario, cells undergoing migration may encounter a different environment from their original location, and hence will respond depending on what new signals are presented to these cells. Lastly, we describe the concept of a transitional matrix which is transiently remodeled, during the process of regeneration, followed by restoration of the native/homeostatic niche, once the entire process is completed.

Quite often these processes cooperate with each other during developmental and regenerative processes, and serve to achieve different outcomes at different stages of these processes. We will address these from the context of musculoskeletal development, repair and regeneration, and cells that contribute to the formation of the skeletal and muscle tissues.

## Fate of a Chondrocyte: The Changing Plasticity of a “Stationary” Cell

The journey of a chondrocyte well illustrates the concept of changing cell plasticity. This cell, derived from the mesodermal lineage is the primary cell for the making of cartilage, a semi-solid hydrated tissue containing mostly of ECM. In development, chondrocytes play key roles in formation of the skeleton. Indeed, the axial and appendicular bones are all formed through the process of endochondral ossification, in which a cartilage template of the future bone is first made, and then are converted into bone. In this journey, the chondrocytes will undergo hypertrophy, and then transdifferentiate into osteoblasts. Further, synovial joints are formed within these cartilage templates that involves a dedifferentiation process, reprogramming chondrocytes back to multipotent progenitor cells within the joint forming region, that will give rise to all the structures of the joint (Wang et al., 2016). Thus, chondrocytes trapped in their own matrix undergo numerous changes, modifying their ECM microenvironment sequentially, providing the niche needed for cell-matrix interaction and availability of signaling molecules for cell maintenance, proliferation, differentiation, dedifferentiation and transdifferentiation.

### Chondrocyte Dedifferentiation in Synovial Joint Formation

During early limb development, condensed mesenchymal forming cartilage templates of the future bones are primed to the chondrocyte lineage, expressing a potent chondrocyte transcription factor Sox9, and producing typical cartilage ECM such as collagen II (Figure 2A). At the site of the future joint (the interzone), changes in molecular signals and ECM occur to facilitate a conversion of the rounded chondrocytes into more flattened interzone cells (Mitrovic, 1978). This process has been described as dedifferentiation as the early “committed chondrocytes” revert to a progenitor status, regaining differentiation potency (Garciadiego-Cazares et al., 2004; Seemann et al., 2005). However, it is also possible that this represents a normal lineage divergence in differentiation in the formation of joints. While the precise signals that drive this process is not clear, patterning genes such as the Hox genes, Wnt and BMP signals are involved (Davis et al., 1995; Storm and Kingsley, 1999; Guo et al., 2004). However, changes in the ECM environment and cell-matrix interaction appear to be sufficient to initiate a joint forming process. For example, inhibition of α5β1 integrin at an ectopic site in a developing limb interrupted cell-matrix interaction of chondroprogenitors, and an ectopic joint is formed at that site (Garciadiego-Cazares et al., 2004). Thus, altering cell-matrix contact is part of the “dedifferentiation” initiation events.

With “dedifferentiation” and formation of the interzone, the ECM environment changes, creating a “softer” structure containing proteoglycans and glycosaminoglycan such as hyaluronic acid (HA), creating an open network structure (Edwards et al., 1994). Binding of HA to its receptor CD44 is essential for normal joint development and cavitation (Dowthwaite et al., 1998). Proliferation of interzone cells are limited, and the interzone expands through further recruitment of the mesenchymal cells from the surrounding mesenchyme (Shwartz et al., 2016). Importantly, the microenvironment is now conducive to direct the differentiation of the incoming mesenchymal cells into interzone cells.

At the molecular level, dedifferentiated interzone cells express high levels of *Gdf5*, and is an earlier gene marker for these cells (Storm and Kingsley, 1999; Seemann et al., 2005), and later, a stem cell marker *Lgr5* (Feng et al., 2019). Cell lineage tracing showed that interzone cells can become cells in the meniscus and cruciate ligaments, and chondrocytes in the articular cartilage (Koyama et al., 2008; Feng et al., 2019). Within the interzone, cells begin to produce a different set of ECM and remodel the microenvironment with the downregulation of *Sox9* and *Col2a1* (Amarilio et al., 2007), and initiate the production of *Cilp* and *Col22a1* (Feng et al., 2019).

Interzone cells at different locations of a developing joint receive their unique differentiation cues and further segregate and differentiate into different compartments. Collagen XXII (*Col22a1*) is a novel maker for late interzone cells as the interzone cells further differentiate to an articular chondrocyte lineage (Feng et al., 2019). Its expression is maintained in the forming meniscus and articular cartilage but absent in the forming cruciate ligaments. The role of collagen XXII in the interzone is not clear, but its expression remains in the most superficial layer of the future mouse articular cartilage (Feng et al., 2019), providing a unique niche for cells residing in this layer. *Col22a1* is expressed at other sites, but limited to cells at tissue junctions or boundaries (Koch et al., 2004). Articular chondrocytes sense changes of mechanical stimuli from the environment and produce *Prg4* (lubricin), an ECM protein that functions to reduce shear at the cartilage joint surfaces (Ogawa et al., 2014). Cellular turnover and ECM remodeling in the articular cartilage is limited, an acceleration of which will cause cartilage destruction, and cellular changes leading to osteoarthritis (van der Kraan and van den Berg, 2012).

### Transdifferentiation of a Chondrocyte to an Osteoblast

The other journey of a chondrocyte in the developing cartilage template is in the process of endochondral ossification (Figure 2B), occurring in the initiation of primary and secondary ossification centers, and establishment the cartilage growth plates at the ends of long bones for linear growth. Thus, articular and growth plate chondrocytes are differentiated through a divergence in their fate (Figure 2C). While they are similarly controlled by the expression of *Sox9* and producing similar ECM at the macroscopic level, they differ in terms of cell state and function that are likely to be controlled by subtle variations at the pericellular level within the different compartments of the articular or growth plate cartilages.

Conceptually, growth plate chondrocytes can be considered as transitional cells for bone elongation, changing and remodeling the ECM in the process. Chondrocytes in the growth plate undergo “scheduled” hypertrophy in this transition to osteoblasts. Interestingly, this is not the case for articular cartilage chondrocytes, except in degenerative conditions such as osteoarthritis (van der Kraan and van den Berg, 2012). Of interest, a recent study showed collagen II suppresses articular chondrocyte hypertrophy and osteoarthritis progression by promoting β1-integrin-SMAD1 interaction, thus inhibiting BMP-SMAD1-mediated chondrocyte hypertrophy (Lian et al., 2019). Therefore, changing the collagen II-containing environment may be a prerequisite condition for hypertrophy, and this is the case in chondrocyte hypertrophy in the growth plate, with the down-regulation of *Col2a1* and up-regulation of *Col10a1*, followed by a complete change in the ECM composition.

The major function of the growth plate cartilage is for the linear growth of long bones. Traditionally, the process of endochondral ossification process has been described in a linear progression, from the activation of chondrocyte proliferation to a hypertrophic stage conducive for mineralization and vascularization (Kronenberg, 2003). The hypertrophic chondrocytes are described as terminally differentiated cells that undergo apoptosis, and the mineralized cartilage matrix is converted to bone, through the action of incoming osteoblasts and progenitor cells. However, this concept has been challenged recently, as a number of cell-fate mapping studies of chondrocytes showed not all the hypertrophic chondrocytes undergo apoptosis, but can transitioned to become osteoblasts, contributing to the bone forming process (Ono et al., 2014; Yang G. et al., 2014; Yang L. et al., 2014; Zhou et al., 2014; Park et al., 2015). This sequence of event indeed demonstrates the plasticity of chondrocytes and hypertrophic chondrocytes that changes in coordination with the ECM. While such changes in plasticity do occur in other systems (Dityatev and Schachner, 2003; Urciuolo et al., 2013; Poltavets et al., 2018), the unique example here is the spatial location of a chondrocyte that is entrapped within a rigid and dense cartilage matrix environment that gradually changes without a physical movement in modifying the ECM to produce a permissive change in the niche of the chondrocytes in the transition process.

### Plasticity Transiting in a Designated Spatial Location

Traditional illustrations show proliferative chondrocytes expanding downwards, and then differentiating into hypertrophic chondrocytes, giving the impression that the cells have moved in their relative spatial position, but in reality, they remained static. In Figure 2D, we present the biological process of endochondral ossification as cartilage is converted to bone, and in the case of a growth plate, there is a continual supply of new cartilage through chondrocyte proliferation, from a switch of “reserved” chondrocytes to a proliferative stage that progresses to the hypertrophic stage (Kronenberg, 2003). Here, there are number of interesting cellular changes from the perspective of ECM cellular states that can be related to the concept of “transition of a stationary cell” (Figure 2D).

In the reserved cartilage, there are chondrocytes embedded in a “sea” of ECM often refer to as rich in collagen II, IX, XI and proteoglycans such as aggrecan, decorin and fibromodulin, and many other cartilage-related ECM including cartilage oligomeric matrix protein (COMP) and matrillins (Myllyharju, 2014). These matrix proteins organize into a functional cartilage structure. Cells within this cartilage are far apart, and the position of the matrix in relation to their distance to the cell and between cells serve different functions. Matrix occupying the space between cells is referred to as the inter-territorial matrix, and is more likely to be structural in nature, whereas matrix closer to each individual is the territorial matrix, linking the inter-territorial matrix to the pericellular matrix (PCM) of the cell, which is immediate to the cell, and presenting as the matrix niche of the cell (Figure 2B).

It was generally accepted that the reserved cartilage contains “quiescent” chondrocytes. Some serve as stem cells, while others closer to the proliferative zone will receive signals for cell cycle reentry for active cell divisions. How these different types of cells are kept in close proximity to each other is not well understood, but likely to fall within the concept of differential microenvironments built within and between chondrocytes. Recent studies have shed light on the potential stem cell niche within the reserved zone in postnatal life (Mizuhashi et al., 2018; Newton et al., 2019). For example, skeletal stem cells appear to be among PTHrP-positive chondrocytes, and PTHrP-positive chondrocytes expressed markers for skeletal stem and progenitor cells (Mizuhashi et al., 2018). In addition, some of these stem cells undergo asymmetric cell division for self-renewal and the other daughter cell contributes to proliferative chondrocytes forming the characteristic columns (Newton et al., 2019). PTHrP-positive chondrocytes response to Indian hedgehog (IHH) signaling, a morphogen gradient that is regulated by HSPG; thus the level of HSPG in the territorial and inter-territorial matrix will have a role.

### Transition to Active Proliferation and Formation of Chondrocyte Columns

While the precise signals that activate the proliferation of chondrocyte forming column of cells is not clear, various growth factors are involved, including mitogenic signals such as insulin like growth factor (IGF), Indian hedgehog (IHH), and anti-mitogenic FGF signaling through FGFR3 (Samsa et al., 2017); all of which are regulated by the ECM in terms of availability and presentation. As indicated earlier, the IHH gradient along the proximal-distal axis of the growth plate, in which heparan sulfate proteoglycans (HSPGs) have a role in establishing this gradient, has differential short- and long-range effects that is concentration dependent, and interacts with the PTHrP/PPR signaling pathway (Kobayashi et al., 2002). Further, membrane bound HSPGs function as a co-factor for FGF receptors in the presentation of FGF ligands to chondrocytes. Indeed, altered range of IHH signaling was observed in mice deficient of *Ext1*, one of the heparan sulfate (HS) polymerizing enzymes, causing growth plate dysmorphology (Koziel et al., 2004).

As the chondrocyte enters the active cell cycle stage, the formation of a column of cells reorganizes the ECM environment. Although it is thought that the overall cartilage component has not changed much, the spatial orientation of the cells in relation to the matrix has changed dramatically, with cell-cell contact along the proximal-distal axis, while there is cell-matrix contact at the peripheral of the column of chondrocytes, entrapped in a cocoon-like structure referred to as a “chondron” (Figure 2B). This is a highly organized and unique structure that is necessary for the elongation of long bones. The importance of chondrocyte stacking is well illustrated in a mouse model lacking β1-integrin (Aszodi et al., 2003), a cell surface receptor necessary for cell-matrix interaction. It is shown that a chondrocyte divides perpendicular to the long axis of the cell, and a mechanical event that allows the sliding of the daughter cells to be stacked along longitudinal axis is controlled by cell-matrix interaction. The stiffer matrix in the longitudinal septum separating the columns restricts the rotating direction of the daughter cells and hence the cell stack formation (Prein et al., 2016). Flattening of proliferating chondrocytes is also correlated with the gradual increase of collagen density and matrix stiffness from embryonic stage to early postnatal stages (Prein et al., 2016).

### Transition to Osteoblasts via Hypertrophy

Hypertrophy of chondrocytes involves a series of phenotypic and cell state changes. At the “prehypertropic” stage, the cells are exiting the cell cycle and the ECM begins to change with down-regulation of much of “cartilage” ECM markers such as *Col2a1* and its transcriptional regulator *Sox9*, while *Col10a1* and *Ihh* expression are increased. Sox9 plays a central regulatory role in chondrocyte hypertrophy. Sox9 has an inhibitory role on *Col10a1* expression in proliferating chondrocytes through the Gli factors (Leung et al., 2011). In early hypertrophic chondrocytes, Sox9 interacts with another transcription factor Mef2c to activate *Col10a1* expression (Dy et al., 2012). However, the ectopic expression of *Sox9* in hypertrophic chondrocytes can suppress *Col10a1* expression (Leung et al., 2011). Members of AP-1 families such as Jun and Fosl2 have also been shown to act together with Sox9 to promote chondrocyte hypertrophy (He et al., 2016). *Col10a1* is a marker for hypertrophic chondrocytes but the function of Collagen X is not clear, as hypertrophy occurs normally in *Col10a1*-null mice with normal growth plate morphology (Kwan et al., 1997). It is proposed to facilitate mineralization of hypertrophic cartilage, ready to be converted to bone (Kwan et al., 1997).

The need for cellular hypertrophy at this stage of endochondral ossification is not clear. A contribution to bone elongation is proposed, and the degree of cell enlargement could be correlated with the rate of bone growth (Cooper et al., 2013). For example, larger hypertrophic chondrocytes were found in rapidly elongating proximal tibia; whereas smaller ones were found in slower elongating proximal radius (Wilsman et al., 1996). However, it is also possible that this change is part of the preparation for the final transitional stage of a chondrocyte to become an osteoblast. Hypertrophy in a confined space requires room for expansion, and thus the need to remodel the ECM for this purpose, and at the same time, could alter the cell-matrix interaction and the cellular architecture in reprogramming the chondrocyte. While the term “hypertrophic chondrocyte” described the origin of these cells, it has none of the molecular characteristics of a chondrocyte; it is a very different cell. In fact, it bears more resemblance with an osteoblast as it expresses *Runx2*, a “master” regulator initiating osteoblast differentiation (Schroeder et al., 2005). Thus, these hypertrophic cells in this new environment is preparing their next journey, and the hypertrophy process alters again the plasticity and the potential of these cells for further dedifferentiation or transdifferentiation.

### End of a Plasticity Journey: The Final Act of a Chondrocyte

The transition of a hypertrophic chondrocyte to an osteoblast was a major discovery that has provided much interest in deciphering the molecular mechanism and the role of hypertrophic chondrocyte in bone formation. This was achieved by using various Cre-drivers marking chondrocytes (Col2a1-Cre) and hypertrophic chondrocytes (Col10a1-Cre) specifically, and follow their fate (Ono et al., 2014; Yang G. et al., 2014; Yang L. et al., 2014; Zhou et al., 2014; Park et al., 2015). While this transition is now well accepted, the mechanism by which this takes place is not clear. However, it is clear that this takes place at the ossification front or the chondro-osseous junction, where there is yet another major switch in ECM, and the environment in which transiting cells encounters. An ECM switch is evident by the high expression level of MMP13 at the chondro-osseous junction, that would actively degrade ECM components (Stickens et al., 2004), and assisted with degradative enzymes from the incoming osteoclasts with the invading vasculature (Gerber et al., 1999), will released the hypertrophic chondrocytes from their enclosed environment of a chondron. This renewed “freedom” sets these cells to reprogram into a multipotent status in cellular plasticity.

This “out of jail” concept is supported by decades-old observations that chondrocyte isolated from their ECM environment, cultured in monolayer dedifferentiate with proliferation and passage; changing morphology and gene expression profiles from *Col2a1*/*Col10a1* expressing cells to mesenchymal like cells expressing *Col1a1* (von der Mark et al., 1977), and stem cell markers (Jiang et al., 2016). Entrapping chondrocytes within a 3D cartilage ECM can maintain the chondrocyte phenotype *in vitro*, and reducing dedifferentiation (Mao et al., 2019), as further support of the role of ECM in the niche. Interestingly, a re-entry into the cell cycle may contribute to reprogramming, inducing the expression mesenchymal stem cell surface marker such as *Sca1* in mice (Park et al., 2015) or zebrafish (Giovannone et al., 2019). Further, osteoblasts and bone marrow adipocytes have been identified as descendants of these Sca1 + cells as evidence for multipotency (Giovannone et al., 2019). In bone fracture repair that involves the formation of a cartilaginous callus, chondrocyte descendent cells marked with *Acan*-CreERT2 and *Col2a1*-CreERT inducible Cre mice showed these cells also transit to become osteoblasts (Zhou et al., 2014; Hu et al., 2017). It was further demonstrated that hypertrophic chondrocytes in the callus transition zone re-entered cell cycles and expressed pluripotency genes such as Sox2, Oct4, and Nanog (Hu et al., 2017).

The long journey of chondrocytes illustrates the relationship between progressive matrix remodeling and cell plasticity (Figures 2A–C). Hypertrophic chondrocytes in this context seem to be highly plastic, acting as progenitors for adipocytes, chondrocytes, osteoblasts, and probably have similar roles at key tissue junction sites, as such cells are found at the insertion site of cartilage endplate to the annulus fibrosus of an intervertebral disc (Roberts et al., 1998), or tendon to bone insert sites (Galatz et al., 2007) where these cell can be identified.

## Changing Plasticity by Migration: Limb Bud Outgrowth

Cells can alter their state through movement out of an old niche and into a new environment, undergoing exposure to new molecular and mechanical signals, and thus new cell behavior. This occurs frequently in the context of the development such that this change in environment is necessary and sufficient for directing the formation of new tissues during embryogenesis. Here, we will discuss events occurring in early limb bud formation to illustrate this concept, where progenitor cells undergo numerous changes and reprogramming for migration, proliferation, and differentiation to the required cell types.

### Key Processes and Compartments of an Early Limb Bud

During limb development, the earliest event is the delamination of cells from the somatopleure epithelium covering the lateral plate mesoderm through epithelial-to-mesenchymal transition (EMT) to form the limb mesenchyme as it buds out (Figure 3A). This process is initiated by transcription factors *Tbx5* (fore limb) and its downstream target, *Fgf10*, whereby FGF10 induces the formation at the ectoderm, the apical ectodermal ridge (AER) that expresses *Fgf8* (Figure 3B). Through a feed-forward relationship between FGF10 and FGF8 signals, the progress zone is established with active proliferation of mesenchymal cells (Crossley and Martin, 1995; Ohuchi et al., 1997). Cells at these early stages in the limb mesenchyme maintain their undifferentiated state and undergo migration and proliferation which contributes to directed outgrowth of the limb bud (Gros and Tabin, 2014; Jin et al., 2019). Thus, the somatopleure cells undergo extensive reprogramming and eventually all change from an epithelial state to a migratory and proliferative state of the mesenchymal cells (Xu et al., 1998, 1999; Gros et al., 2010). As the limb bud grows, these cells will contribute to the skeletal elements of the limb (Zuniga, 2015) while the myogenic precursors for muscle formation migrate in from the myotome (Deries and Thorsteinsdóttir, 2016).

The ECM landscape of the early limb bud dictates how signaling factors such as FGFs and SHH are presented and activated. It also provides a suitable matrix for cells to move and change shape, while suppressing differentiation. Current knowledge shows this is achieved through differential HS sulfation patterns, and enrichment of HA around the progress zone beneath the AER (Kosher and Savage, 1981; Nogami et al., 2004; Li et al., 2007; Figure 3C). Distribution of laminin (Godfrey and Gradall, 1998; Gros and Tabin, 2014) glucosaminoglycans (Li et al., 2007), fibronectin (FN) (Zhu et al., 2020), nidogen (Böse et al., 2006), also support functional compartmentalization of ECM components in the regulation of these early processes.

### Limb Bud Outgrowth: Cell Transformation and Migration Into a New Environment

Somatopleure cells are attached to the epithelium via laminin on the basal side. FGF10 induced EMT is accompanied by the breakdown of the laminin layer (Figure 3D), and dispersal of laminin fragments throughout the limb mesenchyme (Gros and Tabin, 2014). Inhibition of *Tbx5* and *Fgf10* not only inhibits the progression of EMT in the mouse forelimb, but also leads to the accumulation of dense regions of laminin around the somatopleure epithelium (Gros and Tabin, 2014). Detachment of cells through degradation of the laminin enriched layer is necessary for cells to transition from an epithelial phenotype to a mesenchymal migratory phenotype. Once detached, these cells react to signaling cues in the mesenchymal space, leading to migration and proliferation in the limb bud, resulting in expansion of the mesenchyme and formation of the progress zone (Figure 3E). Although laminins have been shown to be involved during later stages of muscle development in the limb (Godfrey and Gradall, 1998), whether these punctate laminin deposits observed at the earlier stages have a direct role in the subsequent proliferation and migration of early mesenchymal limb cells remains to be seen.

### Migration and Proliferation of Cells in the Limb Bud Mesenchyme: Role of Sulfation Patterns on HSPG

Following EMT, the proliferation and migration of cells entering the nascent limb bud continue to rely on FGF signaling in this new environment of the forming mesenchyme (Gros et al., 2010; Gros and Tabin, 2014). FGF signaling is highly dependent on its binding to HSPGs for presentation to the appropriate receptors (reviewed in Matsuo and Kimura-Yoshida, 2013). This is one of the prime examples of how ECM can act to regulate signaling gradients at the cell surface, driving cellular processes and tissue patterning during development. FGF10 and FGF8 target different cell populations with high specificity in the AER and limb mesenchymal cells, respectively. This selective binding of FGF to the appropriate FGF receptor (FGFR) is achieved through HSPG on the cell surface such that FGF10, but not FGF8, will interact with cells at the AER in the zebrafish limb bud (Sun et al., 2002; Norton et al., 2005; Lu et al., 2011). Whereas, CD44 expressed by AER cells binds FGF8 and presenting this to the underlying mesenchymal cells (Sherman et al., 1998; Figure 3E).

The specificity of FGF binding to HSPG is further fine-tuned by the O-sulfation pattern of glucosaminoglycans, achieved through differential expression of sulfate modifying enzymes. In the developing chicken limb bud, it was shown that expression of heparan sulfate 2-O-sulfotransferase (*HS2ST*) and heparan sulfate 6-O-sulfotransferase isoforms (*HS6ST-1* and *-2*) were expressed in different regions with different substrate specificities. *HS2ST* expression was detected throughout the mesenchymal region, with lower expression in the ectoderm in both wing and leg buds. *HS6ST-1* was enriched at the anterior proximal regions of the wing and leg bud, with weak expression at the posterior proximal region. In contrast *HS6ST-2* was mainly expressed in the posterior region of the mesenchyme (Nogami et al., 2004; Figure 3C).

FGF10 requires 6-O-sulfated structures for bind to HSPG, and the expression pattern of the sulfotransferases suggests a higher level of high 6-O-sulfated HSPG in the proximal region than the distal region of the limb bud. This in turn would direct FGF10 signal toward the AER (Nogami et al., 2004). Indeed, functional analysis in chick limb bud development confirmed that FGF10 signaling is dependent on 6-O-sulfated HSPG mediated by *HS6ST-2* (Kobayashi et al., 2010). While the expression pattern of *HS6ST* orthologs (*6OST-1*, *-2*, and *-3*) in the mouse embryo is slightly different to the chick embryo, with *6OST-1* expressed in the mesenchyme and AER, *6OST-2* in the mesenchyme, and *6OST-3* in the distal mesenchyme and epithelium, their expression patterns still consistent with the directed signaling of FGF10 toward the AER (Sedita et al., 2004).

While FGF signaling directs the proximal/distal patterning and outgrowth, the anterior/posterior patterning is controlled another feed-forward loop between FGF4 and SHH signaling in limb development (Laufer et al., 1994). *Shh* is expressed at the zone of polarizing activity (ZPA) located at the anterior region (Figure 3C) that provides a patterning morphogen gradient along the anterior/posterior axis. Here, the activity of SHH is regulated by its binding to 2-O sulfated HSPG as such binding will restrict its cleavage to the soluble form by ADAM17, thereby regulating signaling capacity along this axis in limb patterning such as the number and identity of the digits (Dierker et al., 2009; Figure 3C). Together, these fine-tuning of FGF and SHH signaling by the sulfation pattern of HSPG at the cell surface indicate that cells within the mesenchyme and the AER responds to their environment, and in turn modify its pericellular niche by differential expression of ECM and modifying genes, and cells will act accordingly as they enter into that local environment. In this case, the undifferentiated cells in the progress zone maintain proliferation and migration through exposure to FGFs localized to this region via HSPGs, while also responding to patterning cues from SHH signaling. Differentiation and condensation into skeletal elements initiates when cells leave the influence of this progress zone, which is in part is enabled by changes in hyaluronic acid (HA) levels (Li et al., 2007; Figure 3C).

### An Environment for Cell Proliferation and Migration: Role of Hyaluronic Acid

The ECM within the limb progress zone needs to provide a proper environment for migrating cells to maintain proliferation and their progenitor state. HA as a large unsulfated glycosaminoglycan contributes to this permissive environment. HA is known to regulate cell proliferation and migration (Toole, 2001; Solis et al., 2012), and is produced by the enzyme HA synthase 2 (*Has2*). *Has2* is highly expressed at the distal posterior subridge mesoderm in the chick limb bud, but not in the AER (Li et al., 2007). Thus, there is an increasing gradient of HA in the progress zone along the proximal to distal axis that also correlates with the state of cellular differentiation along this axis (Kosher and Savage, 1981). The lack of HA is suggested to promote physical interaction between the AER and the underlying mesenchymal cells. In addition, CD44 localization in cells of the AER could induce endocytosis and subsequent intracellular degradation of HA (Culty et al., 1992; Hua et al., 1993); thus maintaining a HA-free region between the mesenchymal progress zone and AER (Yu et al., 1996).

Cell proliferation needs a permissive ECM and space, in part related to the stiffness and hydration property of the microenvironment in the limb bud mesenchyme (Wells, 2008; Bott et al., 2010; Notari et al., 2018; Sun et al., 2018). HA could regulate these processes (Li et al., 2007), as it can generate a hydrated PCM with its high affinity for water, separating cells from each other, and providing space needed for changes in cell shape and migration (Toole, 2001). Importantly, HA also has a role in maintaining the limb mesenchymal cells in an undifferentiated state in their journey toward the AER, shielding cells from incoming signals that would otherwise induce a differentiation. Thus with the reducing level of HA and *Has2* expression in the proximal mesenchyme with limb bud outgrowth, precartilage mesenchymal condensations are formed in the proximal central core of the limb bud (Knudson and Toole, 1985; Li et al., 2007). This is consistent with a much-reduced coat of HA around the PCM of cells during mesenchymal condensation, such that cells in the chondrogenic and myogenic regions lacked HA in the PCM (Knudson and Toole, 1985). Further, as in the AER, HA interaction with CD44 at the condensing region will mediated endocytosis of HA leading to degradation of HA, promoting cell-cell contact (Hua et al., 1993; Rousche and Knudson, 2002). Conversely, maintaining Has2 expression will lead to malformation or loss of skeletal elements in the limb (Li et al., 2007).

While HSPG and HA represent the more extensively studied examples of the role of the ECM in regulating the environment in the developing limb, recent studies are beginning to uncover the role of other ECM proteins in limb bud formation and outgrowth such as FN (Zhu et al., 2020) and nidogen (Böse et al., 2006).

### Durotaxis in Limb Bud Formation: Fibronectin and Wnt5a in Establishing a Tissue Stiffness Gradient

Directional migration of cells can act through chemical attractants (chemotaxis) or mechanical stiffness (durotaxis) (Pelham and Wang, 1997). In durotaxis, cells tend to migrate toward stiffer substrates; hence directional movement can be achieved along an increasing stiffness gradient in the tissue controlled by the ECM (Vincent et al., 2013). Recently, a 3D stiffness gradient in the mouse limb bud was revealed, raising the possibility that durotaxis may be involved (Zhu et al., 2020). Interestingly, this corresponded to a domain of Wnt5a-dependent expression of FN and stiffness of the limb mesenchyme. Wnt5a is involved in planar cell polarity (PCP) (Witze et al., 2008) and this would be consistent with a stiffness dependent polarization leading to durotaxis, contributed by a gradient of FN. Thus, a loss of Wnt5a signaling leads to decreased stiffness in the tissue and loss of directional movement (Zhu et al., 2020). A role of the AER is suggested as activation of directional motility toward the AER via Wnt5a signaling acts through FGF8 (Gros et al., 2010). As FN is assembled at the cell surface, with development it is also possible that this Wnt5a/FN/durotaxis relationship continues to function in shaping the limb bud through condensation of precursor sites of skeletal elements and mechanosensing of the cells (Zhu et al., 2020).

### Plasticity of Cells and ECM Dynamics Contributing to Limb Bud Formation

It is clear that the ECM dynamics in this scenario 2 (Figure 1C) is key in controlling cellular plasticity in directing cell movement, maintaining cellular status in the process, and activating differentiation on arrival of their new home. Our review of the early stages of limb bud formation highlighted some of these relationships. However our understanding is limited and needs to be expanded to gain insight into tissue organization, and the spatial relationship of the cells with their own PCM to the structure. The role of additional ECM components need to come into play, such as the importance of nidogen in maintaining basement membrane integrity, an impairment of which leads to altered range of FGF8 signaling from the AER (Böse et al., 2006), or the identification of new differentially expressed ECM genes with developmental time such as *Col12a1* using single cell transcriptomic approaches (Tejedor et al., 2020). Further changes to this ECM at the later stages of limb bud development then facilitate the differentiation of tissues such as cartilage and muscle (Hurle et al., 1994; Godfrey and Gradall, 1998; Arteaga-Solis et al., 2001; McCulloch et al., 2009; Nandadasa et al., 2014).

## Transitional Matrix Enabling Plasticity: The Blastema in Limb Regeneration

**FIGURE 4:**
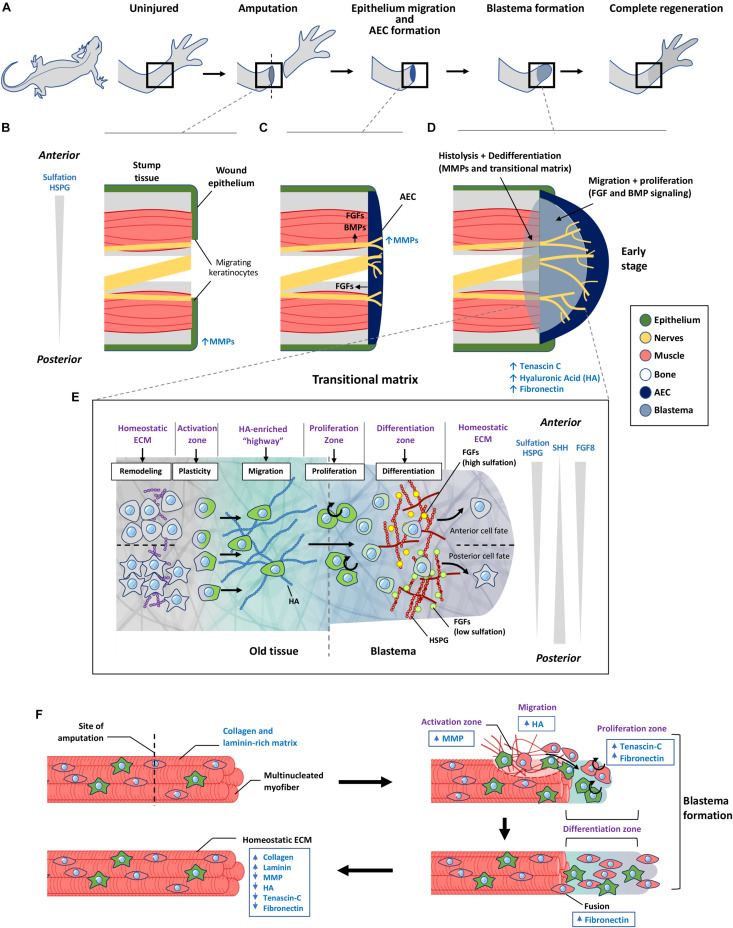
The role of transitional matrix during limb regeneration. **(A)** Overview of limb regeneration. **(B)** After amputation, a wound epithelium is formed through migration of keratinocytes across the wound stump along with the upregulation of MMPs to facilitate degradation of the collagen and laminin-rich ECM. **(C)** As more cells migrate from the epithelium, the wound epithelium is transformed into the multi-cell layered AEC covering the entire wound stump. The AEC and neurons provide essential FGF and BMP signals to initiate blastema formation. MMP expression remains high during this initial stage of blastema formation. **(D)** Formation of the blastema requires remodeling of the ECM to form a transitional matrix. TNC, HA, and FN are upregulated in the distal stump and blastema mesenchyme which enhances dedifferentiation of myofibers, followed by migration and proliferation which depend on FGFs and BMPs to sustain the outgrowth of the blastema. **(E)** ECM regulation of cellular events that occur over during regeneration. Remodeling of the ECM in the old tissue activates cellular reprogramming and migration to form the blastema. Here cells undergo proliferation and differentiation to form the missing tissue. Differences in sulfation levels of HSPG spatially regulate FGF signaling to direct anterior and posterior positional identities. Similar to the limb bud, SHH and FGF8 gradients regulate anterior posterior polarity during later stages of limb regeneration. **(F)** Model of transitional matrix regulation of muscle regeneration. Upon injury, an upregulation of MMPs degrades the native ECM, while upregulation of TNC, HA in the distal stump and blastema promotes dedifferentiation and migration of muscle. Upregulation of FN in the blastema enhances proliferation and fusion of muscle progenitors into myofibers. When regeneration is complete, the transitional matrix components are downregulated, while collagen and laminin is upregulated. The resulting environment therefore returns to the original native ECM of the uninjured muscle. AEC, apical ectodermal cap; HSPG, heparan sulfate proteoglycan; MMP, matrix metalloproteinase; HA, hyaluronic acid.

Regeneration requires detailed coordination of events to guide cells to repair the tissue. Urodeles such as axolotls and newts are able to regenerate complete limbs which makes them an attractive model to study the mechanics of complex tissue regeneration. Inevitably, this process would involve remodeling of the ECM and changes to the signals that cells receive. The existing ECM needs to be altered to form a permissive environment for cells to proliferate, migrate and differentiate in a coordinated manner. While there are many similarities with events during limb development, the regenerative process does not initiate in an embryonic environment and will need to intercalate the old and new tissues. This requires the additional activation of differentiated cells close to the wound, to transiently gain plasticity, proliferative and migratory characteristics. This process can be described through three major events: (1) wound healing and activation of resident cell populations; (2) recruitment and proliferation of progenitor cells to form the blastema; and (3) differentiation and integration of new tissue (Figures 4A–E). During this process a transitional ECM, which is a transient extracellular environment that is different from the native ECM, is remodeled over time to enable different but coordinated cellular processes to occur over the course of regeneration.

### Cellular Landscape of an Early Regenerating Limb

Urodele limb regeneration involves a complex coordination of events that allows for mobilization and activation of cells from the stump tissue (reviewed in Stocum, 2017), which will migrate and accumulate to form a blastema. This blastema grows from recruitment and proliferation of progenitor cells, which then differentiate into tissue cells to build the missing parts, while intercalating with the old tissue to regain a functional limb (Figures 4A–D). The blastema in part resembles the embryonic limb bud as it recapitulates many of the developmental processes of limb formation. However, significant reorganization is needed at the old and new tissue junction to integrate both tissues.

Upon injury, FGF (FGF2 and FGF8) and BMP (BMP2 and BMP7) signals from injured nerves (Makanae et al., 2014) and the apical ectodermal cap (AEC) (Han et al., 2001; Giampaoli et al., 2003), are required for blastema formation, and growth is through activation of cells around the wound site (Figure 4C). There is evidence of conserved signaling pathways activated during limb regeneration. For example in *Xenopus*, the FGF10 and FGF8 signaling loop found in the developing limb bud (Figure 3B), is conserved during limb regeneration (Yokoyama et al., 2000), and FGF10 is also upregulated within 24 h in the axolotl blastema (Knapp et al., 2013). Similarly, FGFs contribute to proliferative cues during regeneration, while SHH is also involved in anterior-posterior patterning during axolotl limb regeneration (Figure 4E; Nacu et al., 2016).

Briefly, migration of epithelial keratinocytes is needed to form the wound epithelium, which then initiates formation of the blastema, initially consisting of primarily fibroblasts of dermal and periskeletal origin. These fibroblasts constitute over 40% of the blastema cell population (Muneoka et al., 1986), and will contribute to connective tissue cells and skeletal morphogenesis (Currie et al., 2016; Gerber et al., 2018). Other lineage restricted progenitors such as mononucleated myoblasts arising from dedifferentiated myofibers, *Pax7* + satellite cells, and neural progenitors also migrate into the blastema to regenerate the missing tissues (Stocum, 2017). This is similar to the embryonic limb bud, where muscle progenitors migrate in from the myotome (Deries and Thorsteinsdóttir, 2016), and skeletal elements differentiate from cells arising from the somatopleure in the limb mesenchyme (Zuniga, 2015). The blastema therefore consists of a heterogonous pool of mainly lineage-restricted progenitor cells that contribute to the different lineages, with dermal and periskeletal fibroblasts possessing a greater level of plasticity as it contributes to both connective tissue and skeletal elements (Gerber et al., 2018). Much like the AER, the AEC is proposed to provide signals that maintain cells in their undifferentiated state while promoting migration and proliferation, while neurons also provide proliferative signals to sustain growth of the blastema (Stocum, 2017).

### ECM Landscape of an Early Regenerating Limb

Like the limb bud, the blastema ECM also has roles in regulation of patterning by providing a permissive environment for cell migration and proliferation to occur, while maintaining cells in an undifferentiated state. Here, we discuss the role of a transitional matrix present at the stump tissue and blastema during muscle regeneration in the limb in regulating the earlier stages of regeneration. Although regeneration of a limb recapitulates many developmental processes, a transitional matrix is needed to enable recruitment of differentiated cells and reprogram them into a progenitor state, which is not required during development. This transitional matrix serves as an “incubator” region for progenitors cells, priming them to the specified lineages (Figures 4D,E). Towards the end of regeneration, the new tissue needs to seamlessly integrate with the original tissue by downregulating components of the transitional matrix. This enables restoration of the native ECM which is described in the example of the regenerating limb muscle (Figure 4F).

### Building the Transitional Matrix at the Wound Site: The Role of MMPs

Initiation of limb regeneration requires formation of a wound epithelium, which is achieved through migration of keratinocytes to cover the amputated limb within 24 h (Figure 4B). Keratinocytes delaminate from the wound epithelium, proliferate and migrate across the injury site over a fibrin and FN network (Donaldson and Mahan, 1983; Donaldson et al., 1989). This requires activation of MMP9 activity (Figure 4B), enabling filopodia formation to occur (Ferris et al., 2010). From this, an AEC is formed that serves similar functions as the limb bud AER, providing essential FGF signals to the underlying blastemal cells (Figure 4C; Campbell and Crews, 2008). A striking difference is the AEC consists of several layers of cells that covers the entire amputated stump, whereas the AER protrudes as a ridge that is located at the dorsal ventral boundary of the limb bud. This difference may reflect the need for a larger number of cells at the AEC to sustain a broader morphogen range and patterning of information to direct the differentiation of cells to build the niche to reconstruct the missing part.

The early AEC lacks an underlying basement membrane structure (Repesh and Oberpriller, 1980; Neufeld and Day, 1996). Thus, facilitating interactions between the AEC and the underlying blastemal cells (Neufeld and Day, 1996). With blastema outgrowth, cells at the AEC express additional MMPs (nCol, MMP9, MMP3/10a, and MMP3/10b), to enable active and efficient ECM remodeling at this critical phase of regeneration (Figure 4C; Vinarsky et al., 2005). Thus, inhibition of MMPs at this stage will lead to failure of blastema formation, as a thick collagen layer under the epithelium impaired the required communication of signals between the AEC with the underlying cells (Vinarsky et al., 2005). Later in regeneration, the AEC begins to build its own ECM niche including FN (Christensen and Tassava, 2000), laminin (Del Rio-Tsonis et al., 1992), collagen IV (Del Rio-Tsonis et al., 1992), and collagen XII (Wei et al., 1995) in its reconstruction of the basement membrane.

MMPs are also activated in the old tissue near the wound site to degrade the existing ECM and to rebuild an integrative boundary between the old and new tissues. MMPs deposited by the wound epithelium and invading macrophages digest the collagen-rich ECM (Figures 4B,C), allowing cells to migrate away from their original environment through a process called histolysis (Godwin et al., 2013; Stocum, 2017). With this, the collagen II matrix in differentiated muscle is reduced (Mailman and Dresden, 1976; Gulati et al., 1983a), as well as major basement membrane components including collagen IV and laminins (Mailman and Dresden, 1976; Gulati et al., 1983a; Rao et al., 2009). The boundary between the old and new tissues is a critical site of the transitional matrix where histolysis and cellular dedifferentiation take place concomitantly (Figure 4D).

Within the old tissue, this change in the ECM environment will alter the niche of specific progenitor cells to proliferate and migrate, or activate dedifferentiation of specific tissue cell types, to be mobilized to build the permissive transitional matrix within the growing blastema. It is also possible that specific MMP-derived cryptic signals from the degradation of ECM molecules could contribute to new cell signaling driving the cellular processes (Schenk and Quaranta, 2003). For example, in the newt limb blastema, the degraded matrix is then replaced by a TNC, FN, and HA-rich matrix (Figures 4D,F; Calve et al., 2010), resembling the limb bud environment that will encourage cellular dedifferentiation, proliferation, and migration.

### Transitional Matrix Enhances Cellular Dedifferentiation, Proliferation, and Migration

FGF and BMP signals presented to the injured tissues initiate blastema formation and blastema growth, which depends on dedifferentiation and recruitment of progenitor cells from the stump tissue into the forming blastema. The properties of the ECM present during this stage is similar in nature to the ECM in the developing limb, providing a hydrated and soft matrix for cells to navigate and maintain their progenitor state at early stages of limb regeneration. Initial activation of myofiber dedifferentiation also resembles the first concept of a stationary cell (Figure 1B) responding to signals and changes that occur in the ECM, resulting in fragmentation and migration out of the old tissue into the blastema (Calve et al., 2010). Here, we provide an example for muscle regeneration where the transitional matrix likely supports plasticity, proliferation, and migration that can apply to other cell types found in the blastema (Calve et al., 2010).

After amputation, the ECM surrounding muscle fibers in the stump region is remodeled from a collagen and laminin rich matrix, to a TNC and HA rich matrix, preceding cellular proliferation with the induction of DNA synthesis (Figure 4F; Calve et al., 2010). TNC is normally localized to defined tissues such as tendons and myotendinous junctions. During regeneration TNC becomes widely distributed throughout the regenerating blastema with a higher level at the distal portion of the stump tissue (Calve et al., 2010). HA is has a similar distribution in a normal blastema (Calve et al., 2010). The dynamic change of HA is similar to limb bud formation, facilitating proliferation and migration. TNC promotes proliferation through its anti-substrate adhesive property as it binds to cells (Murphy-Ullrich, 2001).

TNC can also trigger a series of myogenic programming through two master regulators myoG and myoD, and proliferation through cyclin A upregulation (Fluck et al., 2008). An enrichment of TNC and HA surrounding muscle fibers could therefore support dedifferentiation through fragmentation into mononucleate myoblasts, which then migrate toward the blastema and undergo proliferation (Kumar et al., 2000; Calve et al., 2010; Figure 4F). With dedifferentiation, cells may express embryonic-like antigens that could be targeted by circulating immune cells such as lymphocytes and macrophages. It is thought that deposition of HA in the PCM can prevent an undesired immune response, protects migrating blastemal cells by masking embryonic-like antigens (Alibardi, 2017). As regeneration proceeds to the formation of skeletal elements, both TNC and HA expression is downregulated in regions of condensing cartilage anlagens, allowing cellular aggregation and tissue morphogenesis to be initiated (Calve et al., 2010).

The mechanical property of the transitional matrix is less stiff compared to the stump tissue, that in part contributes myoblast plasticity during regeneration. It has been shown that a soft TNC matrix enhances myofiber fragmentation and migration, while myoblast fusion is promoted in stiffer laminin or FN matrix (Calve and Simon, 2012). FN is less dynamically expressed compared to TNC and HA, but is upregulated together with TNC and HA under the wound epidermis (Christensen and Tassava, 2000; Calve et al., 2010). As regeneration proceeds, there is an increase in FN in the blastema mesenchyme (Gulati et al., 1983b; Nace and Tassava, 1995) which could promote proliferation, and also serve to enhance redifferentiation of myoblasts and fusion to form myotubes (Figure 4F; Calve et al., 2010; Calve and Simon, 2012). Thus, in the limb blastema, the dynamic changes in transitional matrix also fine tunes the mechanical environment contributing to cellular reprogramming and re-differentiation.

### A Return to the “Native” ECM and Homeostatic Cell Niche

As regeneration proceeds, the ECM composition eventually changes to support differentiation and integration of the old and new tissue by a gradual transition back to the original matrix which is indistinguishable from the previously uninjured tissue. For this to occur, inhibition of MMPs in the regenerating tissue is suppressed by the upregulation of tissue inhibitors of metalloproteinases (TIMPS) (Stocum, 2017), together with upregulation of ECM proteins including laminins and other basal lamina components which promotes myoblast differentiation (Calve et al., 2010; Calve and Simon, 2012). Similarly, expressions of TNC and HA are downregulated (Calve and Simon, 2012) such that the muscle microenvironment no longer promotes dedifferentiation, proliferation and migration of cells (Figure 4F). Eventually, plasticity of cells is suppressed as the transitional matrix is further remodeled toward a differentiation inducing environment to complete the regenerative process, resetting all systems back to an original native ECM and homeostatic cell niche (Gulati et al., 1983a; Neufeld and Day, 1996; Mescher, 2004).

## ECM Remodeling During Mammalian Skeletal Muscle Regeneration

Mammalians are unable to fully regenerate limbs, however, there are certain tissues such as the skin, liver, and skeletal muscle that are able to undergo repairs that can be considered as “regeneration.” In mammalian skeletal muscle, regeneration does not involve dedifferentiation, but rather recruitment of muscle progenitor satellite cells (Relaix and Zammit, 2012). Skeletal muscle regeneration is less complex than limb regeneration, but similar concepts apply for the need to recruit progenitor cells through remodeling of the ECM. Here we can observe how each of the 3 concepts described above are applied during this process.

Under homeostatic conditions, satellite cells are kept in a quiescent state until the muscle is damaged and disruption to their surrounding basement membrane leads to activation of these cells (Blanco-Bose et al., 2001; Dumont et al., 2015; Feige et al., 2018; Figure 1B). The ECM niche of satellite cells is located between the sarcolemma and basal lamina of mature muscle fibers (Mauro, 1961). The basal side of the cell attaches to the basal lamina by α7β1 integrin and dystroglycan (Blanco-Bose et al., 2001; Dumont et al., 2015; Feige et al., 2018), which establishes a “polarized niche” that promotes satellite cells to enter a quiescent state (Dumont et al., 2015). Satellite cells contribute to their own niche by depositing basal lamina components such as HSPG Syndecan-3 and Syndecan-4 (Cornelison et al., 2001), which is an example of the stationary cell model (Figure 1B).

Activation of cells to undergo proliferation required remodeling of the ECM, as a form of a simplified transitional matrix (Figure 1D). Here, FGF2 is also involved in directing proliferation of satellite cells, which is regulated by an increase in 6-O and 2-O-sulfation of HS chains, as genetic ablation of HSPGs leads to impaired muscle regeneration in mice (Cornelison et al., 2004). Hence, sulfation of HSPG appears to be a common regulator in activating cells in directing tissue outgrowth, repair and regeneration.

Lastly, differentiation is regulated by the ECM the migrating satellite cell encounters at the site of injury (Figure 1C). Following activation, the muscle progenitor cell migrates along the longitudinal axis of the injured myofiber, along the remnant basal lamina known as a “ghost fiber” (Vracko and Benditt, 1972; Webster et al., 2016) and differentiates into nascent myofibers (Webster et al., 2016). Migrating myoblast-ghost fiber interaction is likely to promote cell fusion into multinucleated fibers, as demonstrated by seeding myoblasts on Matrigel in culture (Melo et al., 1996). Although the detailed mechanism is not known, proteoglycans, FN and laminins are involved in this process (Melo et al., 1996). Disrupted integrin binding to the RGD (arg-gly-asp) domain on FN, collagens, and laminins in culture leads to impaired myoblast fusion (Menko and Boettiger, 1987). These findings are similar to axolotl limb blastema where laminins and FN facilitates formation of new muscle fibers.

This demonstrates how different components of ECM serve to regulate each stage of repair, and that the similarity of ECM proteins involved in both limb and skeletal muscle regenerative processes, including cell activation and differentiation, indicates an evolutionary conserved role of the ECM in controlling the fates of muscle progenitor.

## Discussion

The plasticity of cells is now accepted as a common paradigm whereby the fate of a cell is no longer defined by a differentiated end point, but rather a potentially dynamic state that can be altered given the right microenvironment or niche, to be maintained or changed for a specific outcome. Clearly, our definition of cells need to be revised and as we consider the journey of cells in developmental and regenerative processes, and how they may be recruited in addition to the stem cell pool. The hypertrophic chondrocyte is an example of a cell that may falls into such a category of being capable of existing as a type of “multiple potent progenitor cell.”

While the examples given in this review describe events that happen in different context, in reality, the different scenarios can occur within a specific tissue or different stages of process of development and regeneration. The activation stage depends on local microenvironment changes to activate a local cell population to change, while the migration of cells then brings them into a new environment to proliferate and migrate to the target sites. In regeneration, a transitional matrix is likely to be involved during the entire process, first by creating an environment permissive for proliferation and/or migration, followed by deposition of new ECM that will direct the construction of the missing structures.

The concept of ECM holding positional cues in regulating tissue patterning is exemplified in the axolotl accessory limb model, where the ability to induce the formation and patterning of a blastema on an anterior wound region of the limb by decellularized skin grafts, is dependent on the original anterior or posterior location of the graft (Phan et al., 2015). This in turn is related to the differential sulfation pattern of HSPG present in the anterior or posterior skin grafts, contributing to spatial regulation of FGF signaling (Phan et al., 2015; Figure 4E), analogous to the AER and mesenchymal relationship in normal development of a limb bud (Nogami et al., 2004; Sedita et al., 2004). Interestingly, such positional cues do exist in neonate mammalian skin, such that the graft of a decellularized mouse skin from posterior limb of newborn mice can induce some level of blastema pattern in the accessory limb model that is lost by postnatal day 9 (Phan et al., 2015). Consistently, there is a differential sulfation pattern of HSPG between anterior and posterior skin that is lost by postnatal day 9 (Phan et al., 2015).

Finally, a recent concept of a moving cell population together with a mobile ECM in shaping tissues is intriguing (Loganathan et al., 2016; Hopyan, 2017), raising the potential of need to consider and capture the molecular cues into the world of tissue engineering for repair of regeneration of missing tissues, whereby engineered tissues with cells are implanted, to direct migration of relevant structures together with cells in facilitating the integration and repair process.

Given the ever-growing importance of the ECM, the use of native or biomimetic scaffolds is an area of continuous development, with studies aiming to engineer fully customizable and tunable scaffolds which are biocompatible with native tissues, that can then guide progenitor cells or resident cells toward specific outcomes. Development of tools (Jenkins and Little, 2019), such as electrospinning, 3D bioprinting, lithography, and even a hybrid of different techniques (Dalton et al., 2020), enable the construction of increasingly detailed structures that can better mimic the *in vivo* environment.

For example, scaffolds such as the self-assembling peptide amphiphiles (Sato et al., 2018) developed in recent years is an example of how these scaffolds can be specifically designed to suit regeneration of different tissues such as muscle (Sleep et al., 2017) and nerves (Cheng et al., 2013; Matsuoka et al., 2017). In these studies, peptides mimicking various ECM proteins, such as collagen (Gore et al., 2001; Luo and Tong, 2011), laminin (Cheng et al., 2013), heparin (Mammadov et al., 2011), and TNC (Sever et al., 2014), were incorporated into peptide amphiphiles. These provide signals to cells that interact with this matrix, promoting behaviors such as proliferation and differentiation of cells which aids in repairing tissues such as muscle and can be degraded and remodeled over time with native ECM (Sleep et al., 2017).

Scaffolds have also been designed for bone related regeneration (Donnaloja et al., 2020), which can incorporate key ECM proteins such as collagen (Zhang et al., 2018) and HA (Zhai et al., 2020), or a combination of synthetic polymers with inorganic materials such as hydroxyapatite (Villa et al., 2015) and calcium phosphate (Sauerova et al., 2019). While significant advances have been made over the last two decades, the ideal scaffold for bone tissue regeneration has yet to be developed (Donnaloja et al., 2020). Therefore, a better understanding of the native ECM in bone biology together with improved scaffold fabrication technology will be of importance to make advances in field of bone tissue regeneration (Donnaloja et al., 2020).

The complexity of tissue and cell specific ECM highlights the relevance to precise matching and modifications of ECM proteins required to coordinate and converge mechanical and biochemical signals to the resident cells. It is important to understand how different ECM components function together in future development of biomimetic scaffolds, and to capture the critical cell and ECM information of tissue development and regeneration processes needed for specific temporal and spatial requirements.

## Author Contributions

All authors contributed to the conception, planning, and writing of the manuscript.

## Conflict of Interest

The authors declare that the research was conducted in the absence of any commercial or financial relationships that could be construed as a potential conflict of interest.
